# Machine learning-guided strategies for reaction conditions design and optimization

**DOI:** 10.3762/bjoc.20.212

**Published:** 2024-10-04

**Authors:** Lung-Yi Chen, Yi-Pei Li

**Affiliations:** 1 Department of Chemical Engineering, National Taiwan University, No. 1, Sec. 4, Roosevelt Road, Taipei 10617, Taiwan; 2 Taiwan International Graduate Program on Sustainable Chemical Science and Technology (TIGP-SCST), No. 128, Sec. 2, Academia Road, Taipei 11529, Taiwan

**Keywords:** data preprocessing, reaction conditions prediction, reaction data mining, reaction optimization, reaction representation

## Abstract

This review surveys the recent advances and challenges in predicting and optimizing reaction conditions using machine learning techniques. The paper emphasizes the importance of acquiring and processing large and diverse datasets of chemical reactions, and the use of both global and local models to guide the design of synthetic processes. Global models exploit the information from comprehensive databases to suggest general reaction conditions for new reactions, while local models fine-tune the specific parameters for a given reaction family to improve yield and selectivity. The paper also identifies the current limitations and opportunities in this field, such as the data quality and availability, and the integration of high-throughput experimentation. The paper demonstrates how the combination of chemical engineering, data science, and ML algorithms can enhance the efficiency and effectiveness of reaction conditions design, and enable novel discoveries in synthetic chemistry.

## Introduction

Machine learning (ML) techniques have been widely applied to various chemical-related tasks, such as computer-aided synthesis planning (CASP) [1–4], which can recommend possible synthetic routes for a target molecule and potentially improve the efficiency of developing new synthetic pathways. Many studies have shown that ML-based retrosynthesis models can reproduce patent-derived pathways for known compounds, and even suggest more diverse and efficient alternatives [5–8]. Building upon the retrosynthesis, the reaction conditions prediction models can help in identifying appropriate conditions for each step, ensuring compatibility with the platform and addressing safety concerns. On the other aspect, forward reaction prediction normally plays the role of validating the feasibility of a reaction pathway predicted by retrosynthetic models and to further enhance reaction yields by optimizing reaction parameters such as temperature, pressure, and solvent choice, thus it polishes and trims the suggested routes. As a result, CASP tools have attracted commercial interest and stimulated the development of integrated robotic platforms for automated flow synthesis [9–11].

However, as Coley et al. [12] pointed out, there are still challenges to achieve a fully automated and self-driving synthesis process. One of the key challenges is to automatically select appropriate reaction conditions for each synthesis step without human intervention. Conventionally, the common strategy to determine suitable reaction conditions is to adopt the previously reported conditions for the same or similar reaction types and conduct several experimental trials to evaluate the resulting reaction yields. However, this empirical approach is unlikely to find the optimal conditions, since the reaction outcome depends on a large and complex combination of factors, such as catalysts, solvents, substrate concentrations, and temperature. In academia, especially, the "one factor at a time" (OFAT) approach, which involves changing one factor while keeping the others constant, is frequently used to examine the effect of individual reaction parameters [13]. However, the OFAT method is simplistic and may fail to identify the optimal reaction conditions, since it ignores the possible interactions among the experimental factors.

With the rapid development of high-throughput experimentation (HTE) techniques and ML, it has become more feasible to collect large volumes of data and accelerate the prediction of optimal reaction condition combinations. It has been widely demonstrated that ML algorithms can be used for various chemistry-related tasks, such as yield prediction [14–15], site-selectivity prediction [16–17], reaction conditions recommendation [18], and reaction conditions optimization [13]. These techniques have also been integrated with robotic platforms to speed up the discovery and synthesis of new materials and drug candidates, showcasing the potential and promising benefits of self-driving chemistry labs [19].

Raghavan et al. [20] compared two types of reaction condition models based on their scope of applicability and dataset size: global and local models. The global models cover a wide range of reaction types and typically predict the experimental conditions based on a predefined list derived from literature data. However, this method requires sufficient and diverse reaction data for training, so that the models can have broader applicability and usefulness for CASP in autonomous robotic platforms [12,21]. On the other hand, the local models focus on a single reaction type. Generally, more fine-grained levels of experimental conditions, such as substrate concentrations, bases, and additives, are considered in local models. The development of these models usually involves using HTE [22–24] for efficient data collection, coupled with Bayesian optimization (BO) [25] for searching the best reaction conditions to achieve the desired reaction outcomes.

In this review, we delve into the various methodologies used for predicting and optimizing reaction conditions, and illustrate their diverse applications across different chemical domains. Given the importance of data collection for building data-driven models, we review different aspects of the dataset features and data preprocessing methods. Moreover, we introduce common algorithms and representative studies for developing both global and local models. We highlight representative studies that demonstrate the effectiveness and applicability of these algorithms in real-world chemical scenarios. Finally, we summarize the progress in this field and underline the remaining challenges in the area of reaction condition design.

## Review

### Reaction data collection and preprocessing

One of the major challenges in building ML models for global reaction conditions prediction is the data scarcity and diversity, as they need to cover a vast reaction space [26–27]. However, collecting data relevant to chemical reactions represents a significant challenge. While specific molecular properties can be precisely computed using existing simulation methods like quantum chemical calculations – allowing for the generation of extensive data through large-scale simulations – chemical reactions pose a much greater difficulty for accurate simulation. The development of systematic theoretical calculations to model correlations between reaction yields and various substrates and catalysts requires extensive effort. This involves complex parameter optimization, meticulous validation against experimental data, and careful consideration of diverse reaction conditions and possible reaction mechanisms [28–29]. Although some studies employ transition-state (TS) theory to simulate activation energies and compute reaction enthalpy for particular types of reactions [30], this approach often demands significant computational resources to determine accurate TSs and activation energies. The complexity increases further when considering the impacts of solvents and catalysts, which means that large-scale theoretical calculations are typically restricted to gas-phase reactions [31]. Despite these challenges, recent advances in quantum chemical methods have shown that theoretical calculations can provide practical guidance for validating experimental results [32]. Thus, we posit that the role of theoretical calculations in generating data for ML applications will grow increasingly critical. At present, employing theoretical calculations systematically to construct accurate, large-scale databases of reaction conditions remains highly challenging for complex reaction systems, leading to a primary reliance on experimental data for building ML models.

#### Overview of data sources for chemical reaction modeling

Table 1 summarizes some of the commonly used chemical reaction databases and their characteristics. These databases differ in the types and sources of reactions they contain [33], as well as in the formats used for data recording. Predominantly, these databases rely on experimental chemical data; however, most are proprietary and require subscription-based access. This restricts the availability and comparability of data essential for developing global reaction conditions prediction models and often leads to duplicated efforts in data collection. For instance, Gao et al. [18] trained a reaction conditions recommender on about 10 million reactions from Reaxys [34], but subsequent studies could not access or use the same data for model evaluation or improvement [35]. To address this issue, Coley et al. proposed the Open Reaction Database (ORD) [36], an open-source initiative to collect and standardize chemical synthesis data from various literature sources. The ORD allows chemists to upload reaction data associated with their publications, and aims to serve as a benchmark for ML development. However, the ORD is still in its infancy and contains mostly literature-extracted USPTO data [37], with only a small fraction of manually curated data. Therefore, there is a need for more community involvement and data contribution to make the ORD a comprehensive and reliable resource for global reaction modeling.

**Table 1:** Summary of large-scale chemical reaction databases.

Database	Reference	No. of the reactions	Availability

Reaxys	[34]	≈65 millions	proprietary
ORD	[36]	≈1.7 million reactions from USPTO [37] and ≈91k reactions from the chemical community	open access
Scifinder^n^	[38]	≈150 millions	proprietary
Pistachio	[39]	≈13 millions	proprietary
Spresi	[40]	≈4.6 millions	proprietary

Local reaction datasets, on the other hand, usually focus on a specific reaction family and record reactions with relatively less structural variation in reactants and products. Various combinations of reaction conditions are tested to investigate the output yields in these reaction-specific datasets, which are typically obtained from HTE [41]. Some representative datasets are summarized in Table 2 and can be retrieved from the original papers or ORD. Local reaction datasets have several advantages over global datasets, despite containing less than 10k reactions. For instance, HTE data include failed experiments with zero yields, which are often omitted in large-scale commercial databases that only extract the most successful conditions per reference, as discussed by Chen et al. [42]. This selection bias can lead to overestimation of reaction yields by ML models and limit their generalization capabilities [43]. Therefore, many studies have called for more comprehensive documentation of all experimental results and submission of data in machine-readable formats [44–46]. Another potential issue with data from various sources is the discrepancy in yield definition, as pointed out by Mercado et al. [47]. Literature-extracted yields can be derived from different methods, such as crude yield, isolated yield, quantitative NMR, and liquid chromatography area percentage, and they can also vary in precision due to human bias or equipment quality. HTE data for specific reactions, however, are usually measured using more standardized procedures and are less affected by this issue. In summary, while global models have the appealing feature of wider applicability, local models offer a more practical fit for optimizing real chemical reaction conditions [20]. The choice of datasets depends on the application scenario, whether it is to establish a comprehensive CASP system or to focus on specific reaction types.

**Table 2:** Summary of chemical reaction yield datasets obtained from HTE.

Dataset	Reference	No. of reactions

Buchwald–Hartwig (1)	[48]	4,608
Buchwald–Hartwig (2)	[49]	288
Buchwald–Hartwig (3)	[50]	750
Pd-catalyzed cross-coupling	[49]	1,536
Suzuki–Miyaura coupling (1)	[51]	5,760
Suzuki–Miyaura coupling (2)	[52]	384
Suzuki–Miyaura coupling (3)	[53]	534
electroreductive coupling of alkenyl and benzyl halides	[54]	27
Mizoroki–Heck reaction	[55]	384
coupling of α-carboxyl sp3-carbons with aryl halides	[56]	24
Biginelli condensation	[57]	48
deoxyfluorination	[58]	80
coupling reactions	[59]	264
synthesis of sulfonamide	[60]	39
Ni-catalyzed Suzuki–Miyaura	[61]	450
Mitsunobu reaction	[62]	40
Ni-catalyzed borylation	[63]	1,296
amide coupling (1)	[64]	1,280
amide coupling (2)	[65]	960
Pd-catalysed C–H arylation	[65]	1,536
Ni-catalyzed C–O coupling	[66]	2,003
Ir(I)-catalyzed O–H bond insertion	[67]	653
Pd-catalyzed C–N coupling	[68]	767

Besides the existing datasets, alternative approaches for constructing reaction data through automatic literature mining have also been proposed. These approaches leverage the rapid advancement of natural language processing (NLP) techniques to extract experimental data from unstructured text. For example, Vaucher et al. [69] combined rule-based models and deep-learning techniques to convert experimental procedures into standardized synthetic steps. They further used this data extraction technique to construct a dataset of ≈693k reactions with detailed procedures and developed a sequence-to-sequence model to predict synthetic steps that are actionable and compatible with robotic platforms [70]. Guo et al. [71] conducted a continual pretraining scheme on the BERT model [72] to obtain a domain-adaptive encoder, ChemBERT, which was pretrained on an unlabeled corpus of ≈200k chemical journal articles. They then finetuned ChemBERT on a small annotated dataset for reaction role labeling, resulting in ChemRxnBERT, which can identify the reaction transformation and distinguish reactants, catalysts, solvents, and reagents from chemistry passages. However, many chemical literature records depict reactions using diagrams, which can have various formats such as single-line, multiple-line, tree, and graph representations. Extracting data from reaction diagrams requires the use of image recognition to parse molecular structures and convert them into textual representations. Qian et al. [73–74] demonstrated that this task of optical chemical structure recognition (OCSR) [75] can be handled with a model that combines an image encoder and a molecular graph decoder. Despite the promising machine-learning solutions for reaction diagram parsing [76–77], there are still some limitations. For instance, sometimes the reaction conditions are listed in tables, and certain functional groups in images are represented by abbreviations (e.g., R-groups). To achieve more complete data extraction, future efforts will need to employ multi-modal modeling approaches [78–80] that can collect information from different sources and provide robust results. Recently, Fan et al. developed the OpenChemIE toolkit [81], which integrates extraction methods from text, images, and tables, automating the capture of experimental records of chemical reactions from chemical synthesis papers. This development demonstrates significant advancements in streamlining the data extraction process for chemical research.

#### Implicit data issues and data preprocessing tools

**Figure 1:**
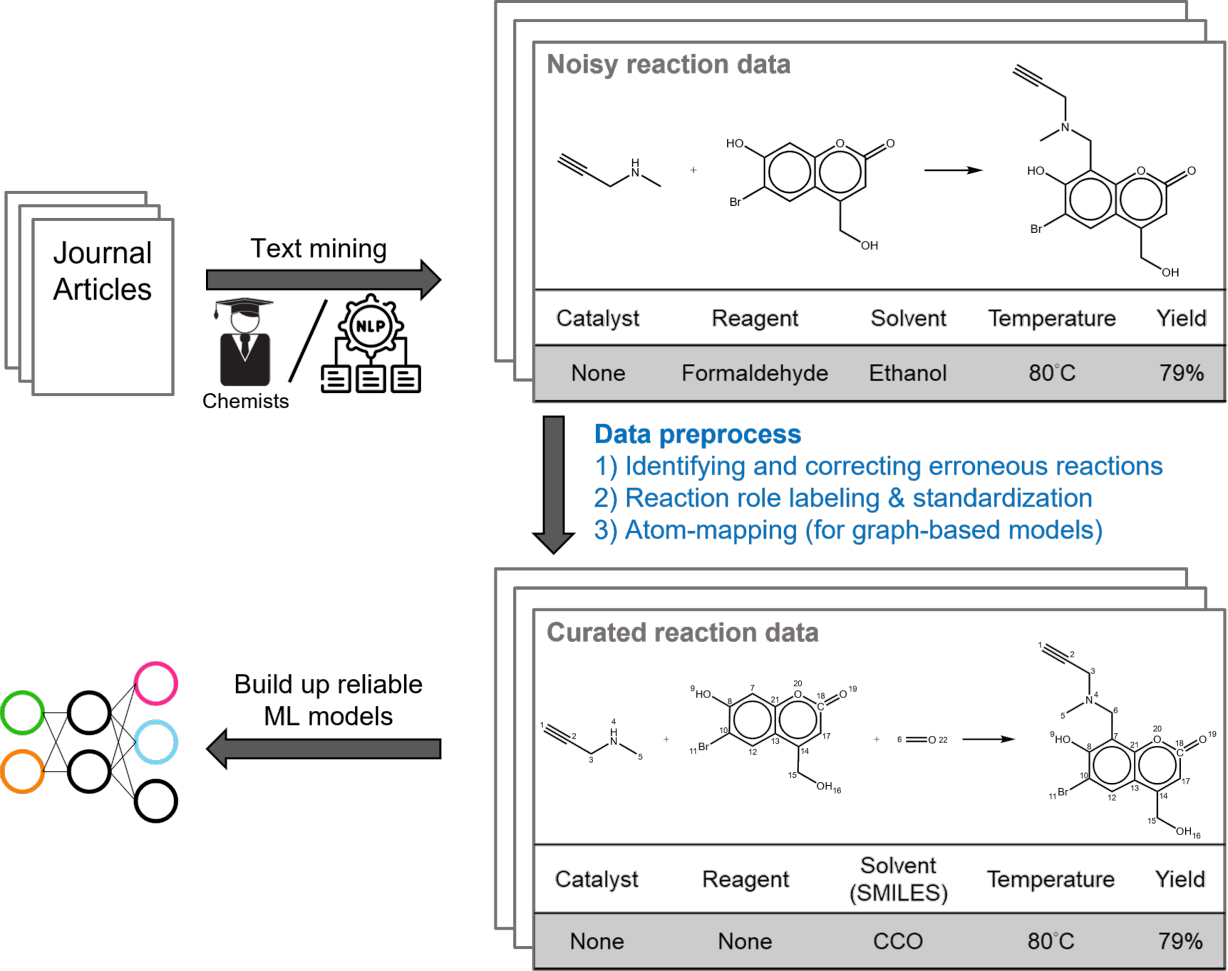
Schematic diagram illustrating the data mining and preprocessing steps for chemical reaction datasets. This process includes data collection, filtering, completion, and atom mapping. In the depicted example, formaldehyde, which contributes a carbon atom to the product, is classified as a reactant due to its active involvement in the reaction. It is also noteworthy that while reaction conditions are ideally encoded in the simplified molecular-input line-entry system (SMILES) to aid in text-based model predictions [82], certain reagents and catalysts, especially those comprising specific mixtures, cannot be represented in SMILES and are instead described using textual labels.

The quality of training data is a crucial factor for the robustness of ML models in chemistry. However, chemical reaction data may contain errors or incompleteness, which can adversely affect the model performance and reliability. The common errors in reaction data can be roughly categorized into two types: (1) erroneous reactions, such as those with mislabeled, missing, or extra atoms in reactants or products, and (2) incomplete reactions, such as those with missing reactants, which are often due to insufficient documentation of the involved species. Erroneous reactions usually require the removal of the corresponding entries from the dataset, as it is hard to determine whether the recorded reactants or products are correct and consistent. Incomplete reactions could be mitigated by using heuristic methods to complete the missing species. In this section, we explain the details of data collection and preprocessing, and we present a schematic representation of the workflow in Figure 1.

One approach to remove erroneous reactions is based on the concept of “catastrophic forgetting”, which refers to the model’s tendency to forget previously learned events during the training process. Toniato et al. [83] proposed to use this idea as a criterion to filter out the reactions that are more difficult for the model to learn, assuming that they are more likely to contain errors. However, this protocol depends on the choice of the model and does not require any chemistry-informed knowledge for preprocessing.

For dealing with incomplete reactions, the first step is to identify the missing component, which can be facilitated by atom-mapping packages [84–87] that assign a unique label to each atom in the reactants and products. With the atom-mapping information, one can apply the rule-based method, CGRTools [88], to add small molecules (e.g., H_2_O and HCl) in reactions, but this method is limited by the availability and coverage of predefined reaction rules. Alternatively, language models have been developed to predict the missing part of molecules given a partial reaction equation, as reported in the work of Zipoli et al. [89] and Zhang et al. [90]. These ML-based approaches can balance reactions without exhaustive rule definition, but they may not be able to recover complex molecules. A promising data preprocessing strategy that addresses this issue is proposed by Phan et al. [91], who formulated the omission of molecules as a maximum common subgraph (MCS) problem and aligned reactants and products to identify non-overlapping segments, thereby generating the missing compounds. Another novel method is AutoTemplate [92], which extracts generic reaction templates from the reactions being preprocessed and recursively applies them on the products of the dataset to validate and correct reaction data. This approach can not only fill in missing reactants, but also fix atom-mapping errors and remove incorrect data entries, thus improving the quality of chemical reaction datasets.

Although many data preprocessing tools have been proposed, we believe more research in this direction can be beneficial to the performance and reliability of machine learning models. Ideally, a unified standard data processing workflow should be established in the future to benefit various reaction prediction and synthesis tasks.

### Reaction representations for reaction modeling

**Figure 2:**
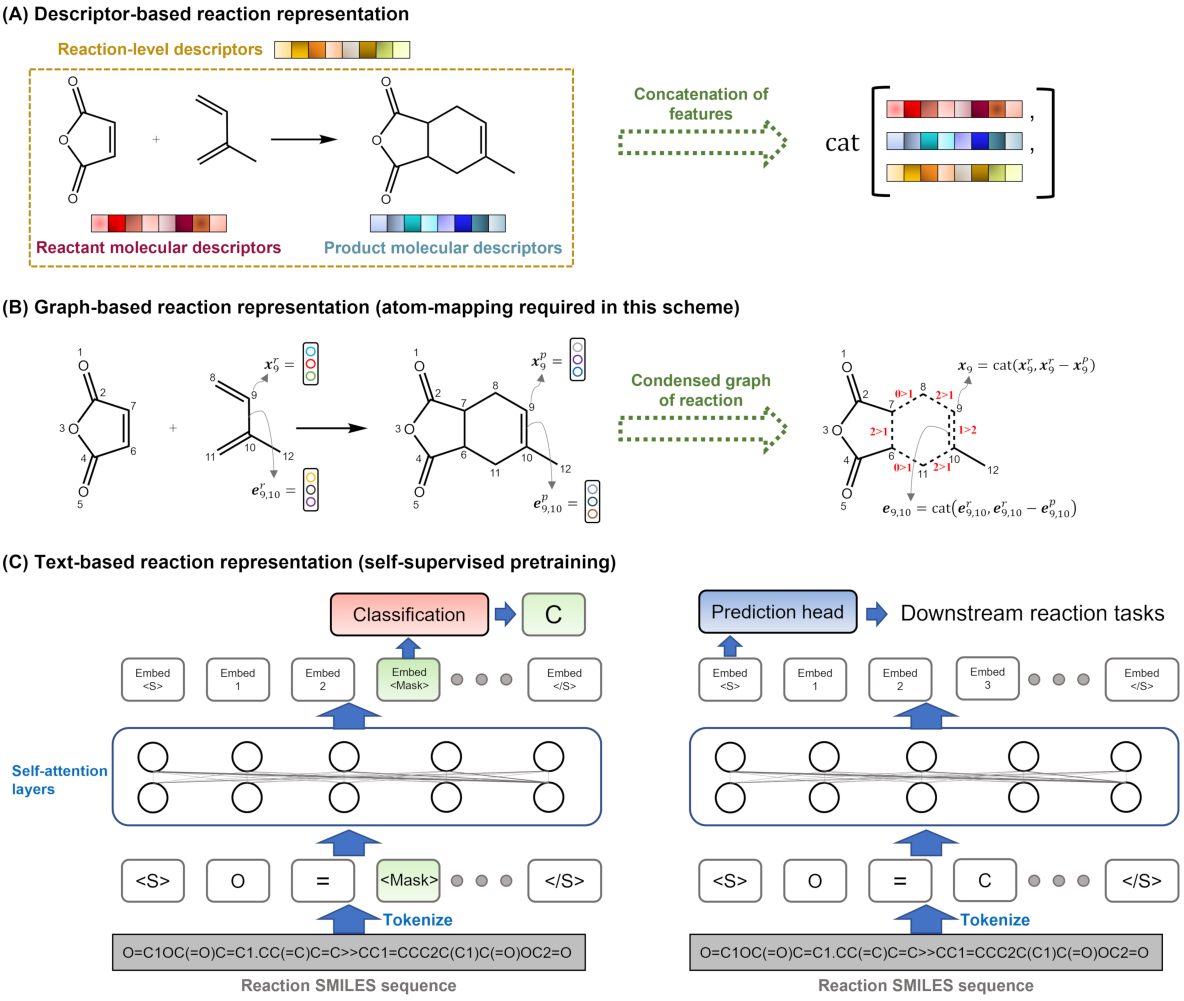
A comparison of three types of reaction embedding methods: (A) descriptor-based, which use predefined features from reactants and products, (B) graph-based, which use neural networks to learn features from molecular graphs, and (C) text-based, which use natural language processing to learn features from reaction SMILES. These methods vary in their computational efficiency, data requirements, and feature interpretability.

The choice of featurization strategy for chemical reactions is crucial for building predictive models for reaction conditions. Compared to the extensive research on molecular representation learning, the development of reaction encoding methods is relatively less explored [93]. Most of the existing methods were originally designed for predicting reaction properties (such as activation energy, reaction enthalpy, etc.) or classifying reactions, but they can be potentially adapted for reaction conditions prediction by modifying the output layer of the model. Both global reaction conditions prediction and local reaction optimization, which use the structures of reactants and products as inputs to predict their corresponding targets, require suitable choices of reaction featurization. The common methods can be categorized into three types: (1) descriptor-based, (2) graph-based, and (3) text-based featurization, as shown in Figure 2. Descriptor-based methods are often used for datasets with limited samples, since they incorporate chemistry- or physics-informed features that can enhance the model's ability to fit the data. Graph-based and text-based methods rely on deep-learning architectures that can learn latent patterns from the reactants and products, but they require sufficient data to train both the feature extractor and the neural network. These methods also reduce the need for manual feature selection by chemists.

#### Descriptor-based representation

Descriptor-based methods are often used for datasets with limited samples, since they incorporate features that are informed by chemistry or physics and that can enhance the model's ability to fit the data [94]. Molecular-level descriptors of reactants and products are concatenated to obtain reaction-level descriptors, which can be computed by various methods [95]. These include substructure keys-based [96–100], circular [101–103], physicochemical [104–107], and quantum mechanical (QM) features [108–112]. The choice of descriptors depends on the size and scope of the dataset. For large-scale global models, descriptors with longer feature lengths and higher computational efficiency, such as the first four methods, are preferred. However, for small-scale local models, QM features can offer more compact and accurate information, but they require sampling and optimizing the 3D conformers of molecules using density functional theory (DFT) calculations, which are computationally expensive and time-consuming [62]. To overcome this challenge, some studies have proposed to pre-generate QM properties datasets and train ML models to serve as fast feature generators for new molecules [16]. However, this approach requires careful validation of the training data coverage and the extrapolation ability of the surrogate models.

Reaction-level descriptors based on DFT calculations of the TS structures of chemical reactions can provide valuable insights for predicting rate constants [113–117], regioselectivity, and site-selectivity [16–17,118–120]. However, this approach is also computationally demanding and requires a good initial guess of the TS structure. Moreover, it may face difficulties in simulating some classes of reactions and large-size molecules [121], and the solvent effects may complicate the results [122]. Therefore, reaction-level DFT-based descriptors are not widely used for reaction featurization. A more popular alternative is the differential reaction fingerprint (DRFP) developed by Probst et al. [123], which converts a reaction SMILES sequence into a binary fingerprint by comparing the symmetric difference of two sets of circular molecular substructures. The DRFP fingerprint can be seen as the reaction version of the ECFP molecular fingerprint [103]. Due to its fast computation and compatibility with conventional ML models, it has been widely used or benchmarked in various reaction-related tasks [124–128], and has become one of the mainstream reaction-level featurization techniques.

#### Graph-based representation

Graph neural networks (GNNs) have been widely applied to various chemical tasks, such as predicting molecular properties [129–133], reaction product prediction [134–136], and inverse materials design [137–139]. Chemical molecules can be naturally represented as undirected graphs, where nodes and edges encode atomic and bond information, respectively. GNNs update and aggregate the hidden features of nodes and edges through recursive message passing and a readout function, resulting in a molecular representation. There are many variants of GNN models [140–143], most of which are based on the message passing neural network (MPNN) framework proposed by Gilmer et al. [144].

Encoding reactions as graph representations is more challenging than encoding molecular structures, as reactions involve multiple disconnected molecular graphs and complex interactions. Graph-based reaction representations can be divided into two categories: AAM-exempted and AAM-required methods. Atom-to-atom mapping (AAM) is a process that establishes the correspondence between atoms before and after a reaction, reflecting the reaction mechanism.

AAM-exempted methods [145–150] apply graph convolutions to each reactant and product molecule separately, and then use a pooling function or attention layers to obtain a reaction fingerprint. These methods are scalable and compatible with conventional GNN models, requiring minimal modifications. AAM-required methods [151–153] assign labels to each atom and adapt the algorithms accordingly. Grambow et al. [151] and Yarish et al. [153] both subtract the hidden node vectors of the reactants from those of the products, and use the resulting differential atomic fingerprints to generate reaction representations. Heid et al. [152] developed a more general AAM-required reaction encoding method that operates graph convolutions on the condensed graph of reaction (CGR) [154–155]. The CGR is the superposition of reactant and product graphs, where nodes and edges can incorporate features from both sides of the reaction, as shown in Figure 2B. This method can also handle imbalanced reactions by imputing or zeroing the missing nodes.

The AAM procedure can provide valuable chemical insights into graph-based reaction encoding, as it reveals how the reaction center atoms influence the bond breaking and formation. However, obtaining accurate AAM for reactions can be difficult and depends on the complexity of the reaction types, as shown by Lin et al. [156]. Moreover, it is unclear whether AAM significantly improves the accuracy of reaction modeling. The AAM-required methods are usually tested on specific reaction types, where the reaction transformations and AAM are clear and correct. However, most large-scale reaction datasets do not have AAM information, and thus require the use of high-accuracy and automated AAM tools [84–87]. These tools may still introduce errors and affect the prediction of new reactions. Therefore, although GNN models are popular and successful for tasks at the molecule level, their effectiveness in reaction-level applications can still be enhanced.

#### Text-based representation

Recent years have witnessed the emergence of large language models (LLMs) [157–159], such as ChatGPT, that learn the statistical and semantic patterns of language through extensive self-supervised training. These models have broad applicability and robust learning capabilities, and thus have attracted the interest of the chemistry domain to tackle relevant problems. One common way to represent chemical molecular structures in chemical databases is the SMILES notation [160], which is a text-based expression with specific grammar rules and can be tokenized as input for language models.

Many studies have adopted the BERT model architecture and the masked language modeling (MLM) method to pretrain on millions of molecular SMILES and finetune on small-sample molecular property datasets [161–164]. For reaction-level prediction tasks, the textual input for pretraining can be changed to reaction SMILES, as shown in Figure 2C. Schwaller et al. [165] first demonstrated this idea and showed that pretraining in this way significantly improved reaction classification accuracy and could automatically generate AAM for reactants and products by analyzing the attention weights of each token in the reaction sequence.

The key to effective language modeling and its powerful reasoning abilities is the size of the pretraining data [166]. However, unlike molecular SMILES, which can be generated from existing databases (e.g., GDB-13 [167]) or by methods that produce reasonable structures [168], reaction SMILES data are often limited by the availability of experimental databases. Therefore, various data augmentation methods [169–171] have been proposed to increase the data size. These methods mainly involve changing the order of SMILES without affecting their molecular structures or modifying specific functional groups in coupling reactions with chemistry-informed reaction templates. Despite the need for large amounts of data to train base models, the main advantage of text-based reaction representation is that it can be easily applied to different downstream tasks by finetuning on small-sample data [172–173], without the need for tedious chemistry-informed feature generation and selection beforehand.

### Reaction conditions design

In this section, we discuss the practical applications of different methods for featurizing reactions in predicting and optimizing reaction conditions. The design of reaction conditions depends on the availability of data and the specific application scenario. For example, if the aim is to predict the reaction conditions for each step in a synthesis pathway as part of an ML-aided CASP system, global models that can handle diverse reactions need to be built using large-scale reaction datasets. These models can then provide a range of general reaction conditions for chemists to select from. Alternatively, if the aim is to optimize the yield and selectivity of a specific reaction, more fine-grained variations of reaction conditions need to be explored. For this purpose, local models that are tailored for specific reaction families need to be trained to provide more focused guidance.

#### Global models for direct reaction conditions predictions

A common approach for chemists to develop novel reactions is to reference similar chemical reactions using reaction similarity search [174–175] and adopt the reaction conditions used in the literature. ML can leverage the large-scale reaction databases to build global models that can predict reaction conditions for diverse and novel chemical reactions, providing initial guidance for chemists.

Most of the existing research on global reaction conditions models involves predicting the reagents used in the dataset as labels, along with the reaction temperatures, using multi-class or multi-label classification methods [176]. This is a convenient way to represent the prediction targets, as some additives, such as molecular sieves and zeolites, cannot be represented by SMILES notation. However, the labels in the datasets may have some inconsistencies, such as different names for the same chemical, which may affect the learning and performance of the models. Therefore, a preprocessing step to standardize the labels and reduce redundancy is also essential.

Gao et al. [18] developed a large-scale model for predicting reaction conditions, using a deep learning approach trained on the Reaxys database. Their model could sequentially predict the catalysts, solvents, and reagents for a given reaction. This approach demonstrated the model's ability to handle complex and diverse datasets. However, the model assumed that each reaction had a single optimal set of conditions, ignoring the fact that some reactions might have multiple viable alternatives. This limitation reduced the diversity of options available for experimentalists. Subsequent studies have attempted to overcome this challenge by proposing different solutions. Kwon et al. [145] used a variational autoencoder (VAE) architecture to sample different reaction conditions, while Chen et al. [42] designed a two-stage recommendation system that predicted and ranked various reaction conditions based on the reaction yields. These methods enabled the prediction of a range of reaction conditions, allowing experimentalists to choose their preferred ones. However, building such a model is difficult, as most reaction databases, such as Reaxys, only record the highest-yield reaction conditions from a single publication. Therefore, the data might lack diversity in reaction conditions for a given reaction, unless the same reaction appears in multiple publications with different conditions.

A variety of ML approaches have been applied to the prediction of reaction conditions, including descriptor-, graph-, and text-based methods, as summarized in Table 3. However, these studies use different reaction datasets to evaluate their models, making it difficult to compare their accuracy objectively. A more standardized and open-source way of storing and accessing chemical reaction data, such as the ORD [36,177] or the curated USPTO dataset [35], would facilitate the benchmarking of models in predicting reaction conditions. Moreover, ML models may not always learn to predict meaningful reaction conditions; they may simply memorize the most frequently reported solvents and reagents in the literature. Beker et al. [178] showed that some machine learning models could not outperform simple statistical analyses based on the popularity of reported conditions in the literature, using the Suzuki–Miyaura coupling as an example. Therefore, to assess the predictive capabilities of models more rigorously, popularity-based baselines should be used as a reference.

**Table 3:** Representative works on predicting globally reaction conditions. The references are sorted chronologically.

Reference	Data	Model type	Description

[18]	≈10 million general reactions from Reaxys	ECFP + DNN	the model has the most access to proprietary training data
[179]	4 types of totally ≈191k reactions from Reaxys	descriptors + GBM and GCNs	the output labels were systematically categorized with chemical insights
[70]	≈693k reactions from Pistachio	nearest-neighbor, transformer and BART	the work demonstrates the first utilization of NLP models to generate the step-by-step experimental procedures
[180]	≈6k Buchwald–Hartwig coupling reactions from in-house lab notebooks	ECFP + DNN	it showed that multi-label predictions are more advantageous than single-label predictions
[145]	4 types of totally ≈191k reactions from Reaxys	GNN + VAE	the models provide multiple reaction conditions by repeatedly sampling from the VAE space
[82]	480k USPTO-MIT dataset [134]	reaction SMILES + transformer	this work directly predicts SMILES representation of the combination of reaction conditions
[35]	curated USPTO-condition dataset with ≈680k reactions and Reaxys-TotalSyn-Condition dataset with ≈180k reactions	reaction SMILES + transformer	this work demonstrates the benefits of MLM pretraining for the downstream reaction conditions prediction task
[42]	10 types of totally ≈74k reactions from Reaxys	ECFP + DNN	it models the reaction conditions prediction problem as recommendation system and artificially generate fake reaction conditions for data augmentation
[181]	curated USPTO-Condition dataset with ≈680k reactions	SMILES-to-text retriever and text-augmented predictor	the two-stage model first uses multimodal retrieval to obtain related chemistry literature and then combines it with reaction input to predict reaction conditions

The choice of reaction conditions is crucial for CASP applications, as it affects the cost, yield, and environmental impact of the synthetic route [4,182]. Moreover, predicting reaction conditions can help optimize the synthetic route [183] by providing the necessary information for each synthetic step. Coley et al. [12] integrated ASKCOS [184], an automated CASP software, with the self-driving lab [185] and demonstrated the synthesis of 15 small molecules. Guo et al. [186] used a synthesis strategy that combines Monte Carlo Tree Search (MCTS) with reinforcement learning to model the retrosynthesis game, aiming to identify high-value synthetic pathways. Recently, Koscher et al. [21] have shown the simultaneous design and synthesis of dye molecules through design–make–test–analyze (DMTA) cycles [187]. Given the limited experimental throughput, it is important to prioritize the molecular properties that are predicted to be superior, along with their synthesis costs, during the chemical experiments. The reaction conditions prediction model plays a vital role in this context; it filters out inaccessible and incompatible conditions, such as high-temperature reactions, high-reactive gases, insoluble solid reagents, and environmentally unfriendly reagents.

The examples above illustrate the usefulness of global reaction conditions prediction models, which use historical literature on similar chemical contexts to suggest suitable reaction conditions for synthetic steps. However, the predictive output often lacks fine-grained details such as reaction time, pressure, and pH values. These details depend on the problem formulation specific to each individual synthetic step. To further improve yields, it is necessary to perform local reaction optimization, which is discussed below.

#### Local reaction optimization

**Figure 3:**
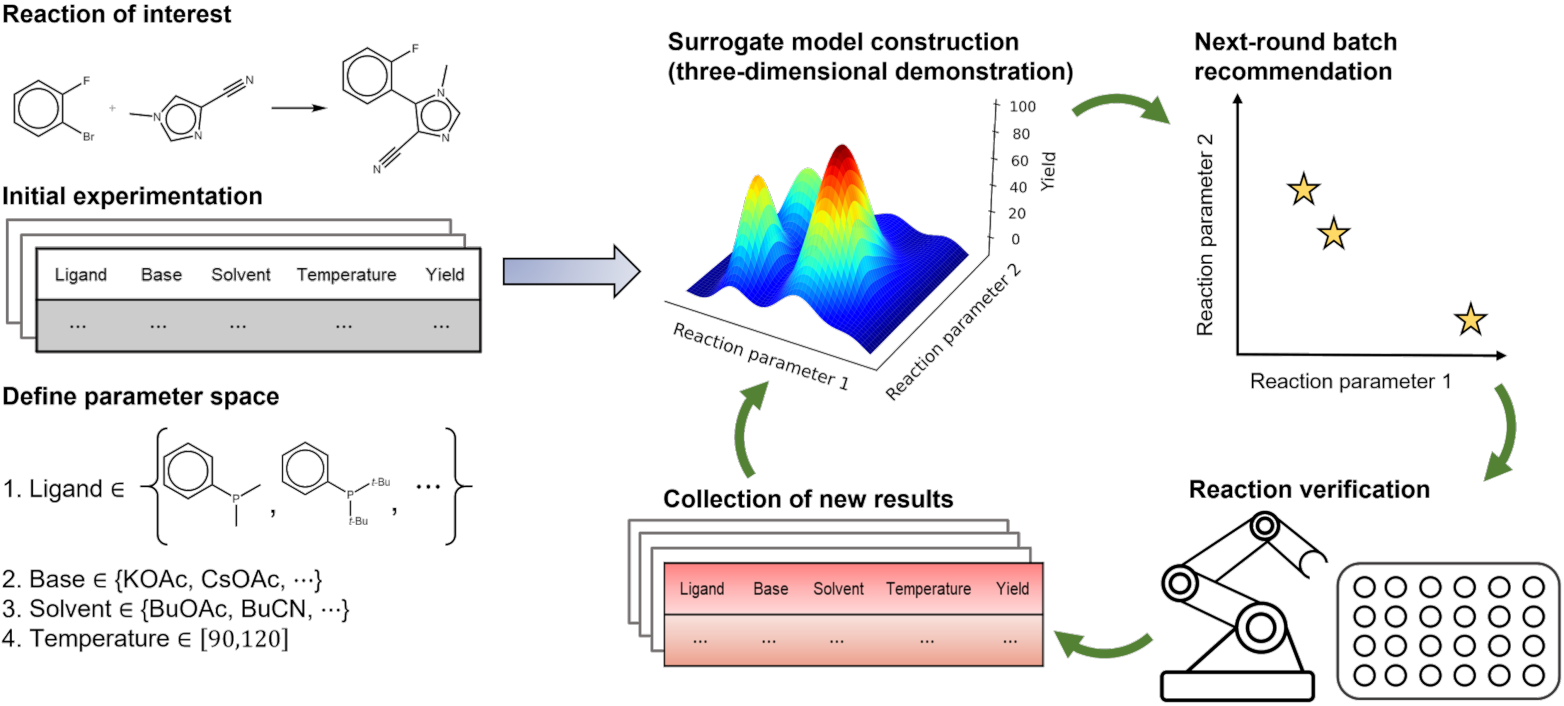
A schematic diagram of how ML algorithms can be combined with HTE platforms to optimize reaction conditions for CASP.

ML-guided local reaction optimization, or self-optimization, is an automated and generalizable approach that can accelerate the discovery of optimal reaction conditions, as illustrated in Figure 3. The first step is problem formulation, which involves defining the reaction parameters to be optimized and the target objectives, such as yield and selectivity. The reaction parameters include categorical variables, such as catalysts, solvents, and acid–base salts, and continuous variables, such as temperature, pressure, substrate concentration, and residence time. Regression prediction models are then built for these reaction parameters and target objectives by collecting experimental data and conducting statistical analysis.

Many reaction optimization platforms have been developed [188–191], which integrate software optimization algorithms with hardware automation for experiments, enabling large-scale experimentation and data collection. Among these, BO [192] is the most classic and widely used algorithm, which leverages kernel density estimators to efficiently explore parameter space. This method updates prior probability distributions with new experimental results and optimizes the reaction conditions by focusing on regions of the parameter space predicted to improve objectives. The power of BO lies in its ability to balance exploration and exploitation, making it highly effective for complex, multidimensional optimization tasks in chemical processes. BO has also demonstrated robust performance in many benchmark tasks [193–195], and numerous chemical reaction optimization packages have been developed to support this algorithm [196–200].

A typical example is the work by Shields et al. [62], who used different featurization strategies, such as DFT [108], cheminformatics [107], and binary one-hot-encoded, in conjunction with the BO algorithm to optimize reaction conditions. Their experimental results showed that DFT features could train probabilistic surrogate models more effectively and that the optimization efficiency was superior to manual adjustments made by professional chemists. They also applied this approach to the Mitsunobu reaction and deoxyfluorination reaction, rapidly identifying medium to high-yield results from approximately 100,000 experimental conditions using fewer than 100 experiments.

Moving from individual synthetic steps to CASP, Nambiar et al. [201] investigated the impact of integrating a global reaction conditions prediction model with local reaction optimization on enhancing the overall chemical synthesis pathway. They demonstrated the predictive pathway for sonidegib synthesis, but it still required chemical insights to verify the compatibility of the solvents predicted by the global model with the reactants. Moreover, in a multistep synthesis route, the interdependencies between different reaction sequences, such as additional separation and purification steps, could reduce the overall yield [202]. This indicates that the suboptimal combination of each reaction does not necessarily represent the global optimum for multistep synthesis [203–205]. In contrast, telescoped flow sequences [206–208] or one-pot batch synthesis [209] emphasize the use of chemically compatible reagents and solvents in each reaction step to minimize intermediate purification steps. Volk et al. [210] developed AlphaFlow, which utilizes reinforcement learning as an optimization algorithm for the shell growth of core-shell semiconductor nanoparticles. This involves various unit operations such as phase separation, washing, and continuous in-situ spectral monitoring. Although the process conditions for this reaction system do not have as extensive a literature base for training data, this study was still able to identify better solutions than conventional designs through reinforcement learning in multistep processes.

Besides maximizing the reaction yield for a given reaction with given substrates, another goal of reaction optimization is to discover general reaction conditions that are applicable to various substrates within the same reaction type [211–215]. For instance, the generality of chiral catalysts for asymmetric or enantioselective catalysis has been a longstanding interest in synthetic chemistry [216]. Angello et al. [53] applied uncertainty-minimizing ML and automated robotic experimentation to accelerate the exploration of general reaction conditions for heteroaryl Suzuki–Miyaura cross-coupling. They achieved an average yield that was twice as high as that of previous human-guided experiments. Recently, Wang et al. [65] formulated the optimization of general reaction conditions as a multi-armed bandit problem, where each set of reaction conditions is a slot machine, and each experiment is a round of playing on one of these machines. The challenge is to find the slot machine with the highest win rate using a limited number of rounds. For chemical experiments, this entails a strategic balance between exploring new reaction conditions (or 'slot machines') and exploiting known conditions that deliver high yields. Therefore, they proposed a more efficient sampling strategy based on reinforcement learning to dynamically adjust the selection process, thereby optimizing the exploration–exploitation trade-off.

The preceding examples demonstrate how the combination of HTE chemistry tools and optimization algorithms has significantly advanced the field of reaction optimization. However, this protocol also has some limitations, especially regarding the suitability of the chemical system under investigation. First, in terms of hardware implementation, setting up an HTE platform with robotic technologies entails high financial costs and specialized knowledge for installation, which may not be accessible for smaller-scale or less-funded research entities [217]. Moreover, to enable experimentation with various reaction conditions, a large chemical storage capacity is necessary. Otherwise, the scope of research would be confined to only a few types of chemical reactions [21]. Additionally, to ensure experimental safety, chemists must rigorously verify the compatibility of each solvent and reagent combination used in reactions and eliminate any potential hazards [218]. Second, in terms of algorithmic approaches, the widely used BO requires initial data to build a probabilistic surrogate model. Although the data might be sourced from related literature, caution is advised as experimental apparatus from different sources could introduce systematic errors in reported yields [46]. Furthermore, BO cannot generalize well from past reactions to unseen reaction transformations, which inherently requires gathering new relevant data for new chemical reactions [219]. Regarding general reaction conditions, the typically limited experimental budgets in laboratories restrict the ability to explore a diverse range of reaction conditions [65]. Thus, initial filtering by chemists, which removes known impractical conditions, is essential. Despite these existing challenges, reaction optimization continues to play a vital role in both academia and industry in the age of big data [23].

## Outlook and Perspectives

As we explore the future of ML in designing and optimizing reaction conditions, several promising avenues and challenges are poised to shape this interdisciplinary field. The integration of HTE with ML is revolutionizing how chemists approach reaction conditions. Future efforts should aim to enhance these technologies to enable faster and more comprehensive data collection, potentially leading to the automation of HTE and ML integration into real-time adaptive systems that learn from each experiment.

The discussions in this review about global and local models underline the critical need for large, comprehensive, and coherent datasets. Advancements in data processing and model training methodologies, such as transfer learning and reinforcement learning, are essential to boost the predictive power and efficiency of these models. Platforms like the ORD are crucial in meeting the demand for accessible and standardized chemical data. The expansion of such platforms and fostering wider community involvement will be key to advancing data-driven approaches in chemistry. A community dedicated to openly sharing data and findings will likely accelerate innovation and enhance the robustness of ML tools.

Moreover, computational models that integrate theoretical chemistry and ML could unlock deeper insights into poorly understood or complex reaction mechanisms. These models are particularly valuable in areas where experimental data are sparse or challenging to obtain, thereby extending the range of ML applications in chemistry.

Educating the next generation of chemists, engineers, and data scientists in both ML and chemical synthesis is critical. Interdisciplinary programs can develop a workforce skilled in applying AI to complex chemical issues, fostering more innovative and efficient solutions. Enhancing international cooperation can standardize data collection and sharing practices, simplifying the process of building and validating models across various laboratories and contexts. Such global collaboration is instrumental in addressing widespread challenges like climate change and sustainability through smarter chemical processes.

By focusing on these directions, we anticipate a future where ML not only supports but significantly propels the field of synthetic chemistry forward, making it more innovative, efficient, and sustainable. The ongoing development of ML in reaction conditions design and optimization holds the promise of unlocking new capabilities and achieving transformative breakthroughs in the field.

## Data Availability

Data sharing is not applicable as no new data was generated or analyzed in this study.
